# Novel Role for γ-Catenin in the Regulation of Cancer Cell Migration via the Induction of Hepatocyte Growth Factor Activator Inhibitor Type 1 (HAI-1)*[Fn FN2]

**DOI:** 10.1074/jbc.M114.631820

**Published:** 2015-04-29

**Authors:** Marybeth Sechler, Stanley Borowicz, Michelle Van Scoyk, Sreedevi Avasarala, Sereke Zerayesus, Michael G. Edwards, Manoj Kumar Karuppusamy Rathinam, Xiangmin Zhao, Pei-Ying Wu, Ke Tang, Rama Kamesh Bikkavilli, Robert A. Winn

**Affiliations:** From the ‡ Cancer Biology Program and; ‖ School of Medicine, Division of Pulmonary Sciences and Critical Care Medicine, University of Colorado Anschutz Medical Campus, Aurora, Colorado 80045,; § Division of Hematology and Oncology and; ¶ Division of Pulmonary, Critical Care, Sleep and Allergy, Department of Medicine, University of Illinois at Chicago, Chicago, Illinois 60612, and; ** Jesse Brown Veterans Affairs Medical Center, Chicago, Illinois 60612

## Abstract

γ-catenin (Plakoglobin), a well-described structural protein functioning at the adherens junctions and desmosomes, was shown to be either lost or weakly expressed in non-small cell lung cancer (NSCLC) cells and tumor tissues. However, the tumor suppressive affects of γ-catenin were not fully understood. In this study, we have identified a novel role for the affects of γ-catenin on non-small cell lung cancer (NSCLC) cell migration. Expression of γ-catenin in NSCLC cells resulted in reduced cell migration as determined by both scratch assays and trans-well cell migration assays. Moreover, the affects of γ-catenin on cell migration were observed to be p53-dependent. Mechanistically, the anti-migratory effects seen via γ-catenin were driven by the expression of hepatocyte growth factor activator inhibitor Type I (HAI-1 or SPINT-1), an upstream inhibitor of the c-MET signaling pathway. Furthermore, the re-expression of γ-catenin sensitized NSCLC cells to c-MET inhibitor-mediated growth inhibition. Taken together, we identify γ-catenin as a novel regulator of HAI-1, which is a critical regulator of HGF/c-MET signaling. Therefore, targeting γ-catenin-mediated HAI-1 expression might be a useful strategy to sensitize NSCLC to c-MET inhibitors.

## Teaser

**Background:** γ-Catenin is a tumor suppressor in lung cancer.

**Results:** γ-Catenin is a novel regulator of HAI-1 and cell migration.

**Conclusion:** γ-Catenin and HAI-1 regulate cell migration and sensitize NSCLC cells to c-MET inhibitor.

**Significance:** Targeting γ-catenin-mediated HAI-1 expression might be a new strategy to treat lung cancer.

## Introduction

Lung cancer is the leading cause of cancer mortality in the United States (1, 2). The large number of mortalities is in part due to lack of early detection interventions, resistance to existing therapies, and disease metastasis. Although targeted therapies have shown some promise (3), these therapies are restricted to limited cases due to infrequently characterized driver mutations (3). Therefore identification of novel regulators of key signaling pathways that are frequently de-regulated in lung cancer are needed for developing new therapeutic targets.

One signaling pathway that has been a focus of active research in lung cancer is the c-MET signaling pathway (3–6). The c-MET signaling has been shown to play an important role in cell proliferation, survival, and motility (3–6). The c-MET signaling is initiated upon binding of the hepatocyte growth factor (HGF)^2^ to the MET receptor. HGF binding to the MET receptor causes downstream activation of the PI3K/Akt and MAPK signaling pathways, resulting in cell survival, proliferation, and motility (6, 7). A key regulator of c-MET receptor activation is the hepatocyte growth factor activator inhibitor type 1 (HAI-1 a.k.a SPINT-1). HAI-1 is a transmembrane serine protease inhibitor that contains two extracellular Kunitz domains, with its N-terminal KD1 domain responsible for binding to and inhibiting hepatocyte growth factor activator (HGFA) (8, 9). HGFA, another serine protease member, is required for cleavage and activation of pro-HGF (10–14). Despite such tight control, aberrant c-MET signaling has been implicated in several malignancies, including lung cancer (5, 16). In this study we have identified plakoglobin (γ-catenin) as a novel regulator of HAI-1 expression.

Plakoglobin (γ-catenin) is a member of the armadillo repeats containing proteins (17) that exhibits diverse cellular functions including structural roles as well as transcriptional regulatory roles (18, 19). Some of the structural roles of γ-catenin include linking the cytoplasmic portions of cadherins to actin microfilaments and α-catenins in the adherens junctions and linking the cadherins, desmoglein, and desmocolin to the intermediate filaments in the desmosomes (20). Interestingly, loss of γ-catenin has been associated with shorter disease-free survival and worse overall survival in non-small cell lung cancer (NSCLC), particularly in early-stages of the disease (21). Earlier studies have also demonstrated that γ-catenin was weakly expressed or absent in several NSCLC cell lines and that restoration of γ-catenin in these cell lines was observed to be anti-proliferative (22). Furthermore, expression of γ-catenin in SCC-9 squamous carcinoma cells induced a mesenchymal to epidermoid phenotype (23).

In the current study we have identified a novel role for γ-catenin in the regulation of cell migration, which is an important step for tumor progression and metastasis. Interestingly, re-expression of γ-catenin in NSCLC cell lines resulted in reduced cell migration as determined by both scratch and trans-well cell migration assays. Additionally, we demonstrate that the γ-catenin-induced anti-migratory effects were mediated via the expression of HAI-1 in a p53-dependent manner. Taken together, γ-catenin is shown to be a novel regulator of HAI-1 that is a critical regulator of HGF/c-MET signaling. Therefore targeting γ-catenin-mediated HAI-1 expression might be a useful strategy to sensitize NSCLC to c-MET inhibitors.

## Experimental Procedures

### 

#### 

##### Cell Culture

Human non-transformed lung epithelial (Beas2B) cells and the NSCLC cell lines (H157, H1299, and A549) were obtained from the tissue culture core of the University of Colorado, Anschutz Medical Campus. All cell lines were cultured in RPMI 1640 medium (10–040-CV, Cellgro, Mediatech Inc., Manassas, VA) supplemented with 10% fetal bovine serum (FBS) in a humidified 5% CO_2_ incubator at 37 °C. Cell lines were cultured bi-weekly and stocks of cell lines were passaged no more than ten times for use in experiments. H157 and A549 cells stably expressing pLNCX and pLNCX-γ-catenin plasmids were developed as described earlier (22).

##### Knock-down Protocol

Double-stranded RNAs (siRNAs) targeting human γ-catenin (CCCUCGUGCAGAUCAUGCGUAACUA) were procured from Invitrogen. Control siRNA (sc-37007), p53 siRNA (sc-29435), and HAI-1 siRNA (sc-39554) were purchased from Santa Cruz Biotechnology. NSCLC cells were treated with 50 nm siRNAs by using Lipofectamine 2000 reagent according to the manufacturer's protocol. p53 shRNA construct in TRC1 pLKO-puro backbone was obtained from Dr. Chris Porter (University of Colorado, Anschutz Medical Campus) and was used to transfect NSCLC cells with Lipofectamine reagent as per the manufacturer's recommendation.

##### Transfections and Luciferase Reporter Assays

The reporter plasmid (p53-RE-luciferase reporter), expression plasmids (pcDNA3.1, FLAG-γ-catenin, WTp53), or siRNA (Control siRNA-A, p53 siRNA, γ-catenin siRNA) and CMV-β-galactosidase control plasmids were transiently transfected into cells using Lipofectamine 2000 Reagent (11668019, Invitrogen) as per manufacturer's recommendations. p53-luciferase reporter, with luciferase under the control of 14 repeats of p53-binding sequence (TGCCTGGACTTGCCTGG) was obtained from Stratagene. After 24 h, the lysates were assayed for luciferase activities. The luciferase values were normalized to β-galactosidase values and are represented in the graphs.

##### Immunoblot Analysis

Cell extracts were prepared in a lysis buffer (0.5% Triton X-100, 50 mm β-glycerophosphate, pH 7.2, 0.1 mm sodium vanadate, 2 mm MgCl_2_, 1 mm EGTA, 1 mm dithiothreitol, 2 μg/ml leupeptin, and 4 μg/ml aprotinin) and the Western blot analysis was carried out as previously described (24). The following antibodies were used for immunoblotting: γ-catenin (610253, BD Biosciences), HAI-1 (H-1, sc-137159, Santa Cruz Biotechnology), p53 (2524, Cell Signaling Technology), and actin (A3853, Sigma-Aldrich). Aliquots of various protein extracts were resolved on 10% SDS-PAGE gels and transferred to nitrocellulose. The filters were blocked in Tris-buffered saline (10 mm Tris-HCl, pH 7.4, 140 mm NaCl, containing 0.1% Tween-20 (TTBS) and 3% nonfat dry milk and then incubated with the same blocking solution containing the indicated antibodies at 0.5 μg/ml for 16 h. Filters were extensively washed in TTBS, and bound antibodies were visualized with horseradish peroxidase (HRP)-coupled secondary antibodies.

##### Cell Proliferation Studies

Clonogenic assays were performed in triplicates by seeding 1000 cells per well in a 12-well culture plate followed by incubation at 37 °C in a 5% CO_2_ incubator. After 5–7 days colonies were stained using a staining solution (0.5% Crystal Violet, 12% glutaraldehyde, 87.5% H_2_O) for 1 h at room temperature. After de-staining in water and drying, colonies were quantified using Bio-Rad Chemidoc Imaging System and Quantity One Software. Cloning efficiency represents the mean number of colonies formed per well.

MTS cell growth assays were performed in triplicates by seeding 500 cells per well in a 96-well culture plate, followed by incubation at 37 °C in a 5% CO_2_ incubator. Cell proliferation was measured at 24, 48, and 72 h by adding 20 μl of MTS reagent (Cell Titer 96® Aqueous One Solution, G3582, Promega, Madison, WI) to each well, followed by incubation at 37 °C. After 1 h, the absorbance of the formazan product was measured at 490 nm using a plate reader. Normalized absorbance values (sample readings-readings of medium only blank) were represented in the graphs.

##### Inhibitor Studies

For the measurement of cell growth rates, H157 cells (10,000/well) were seeded in a 24-well culture plate. After 24 h, the cells were treated with either vehicle control (DMSO), 5-Aza (3 μm), Tivantinib (0.5 μm), or 5-Aza + Tivantinib. After 48 h of treatment, cells were trypsinized from the wells with 100 μl of trypsin, diluted with 400 μl of growth medium, and counted using a hemocytometer.

Similarly, H157 cells stably expressing either empty vector or γ-catenin were seeded in a 24-well plate (25,000/well). After 24 h, the cells were treated with either vehicle control (DMSO), or Tivantinib (0.5 μm). On subsequent days, cells were trypsinized from the wells with 100 μl of trypsin, diluted with 400 μl of growth medium, and counted using a hemocytometer.

##### Scratch Assay

Cells were grown to confluence in complete cell culture medium. At time zero, a 3-mm scrape wound was created across the diameter with a pipette tip followed by extensive washes with medium to remove dead and floating cells. After adding complete medium supplemented with 1 μg/ml Mitomycin C, cell migration was recorded at 0, 12, 24, and 48 h. Images were captured using an inverted microscope equipped with a digital camera. Images were later analyzed by determining the distance between the cells on either side of the scratch overtime, and are represented in the figures as percent scratch closure.

##### Cell Migration Assay

For assessing cell migration, 30,000 cells in serum free media were seeded into the trans-well inserts (Corning) containing 8 μm permeable pores and allowed to migrate toward 10% FBS containing medium. Later, the cells in the trans-well inserts were removed and the inserts were washed in PBS for three times. The migrated cells on the bottom of the insert were fixed with 2% glutaraldehyde solution followed by crystal violet (1%) staining. After washing the inserts three times with PBS, the inserts were allowed to air dry and pictures were taken using an inverted microscope. Ten independent fields were counted for each trans-well and the average number of cells/field were represented in the graphs.

##### Annexin V Apoptosis Detection by Flow Cytometry

For measuring apoptosis, FITC Annexin V apoptosis detection kit I was used (556547, BD Pharmingen, San Jose, CA). Briefly, H157 cells stably expressing γ-catenin were trypsinized, and washed three times with PBS. Later, cells were stained with Annexin V according to manufacturer's protocol. Flow cytometry was performed using a Beckman Coulter Gallios (Beckman Coulter, Pasadena, CA) flow cytometer at the UIC flow cytometry core facility.

##### Pearson Correlation Studies

Lung cancer cell line gene expression datasets from the Cancer Cell Line Encyclopedia (CCLE) (GEO#GSE36133, 166 unique cell lines) and UT Southwestern Medical Center (GEO#GSE4824, 76 unique cell lines) were downloaded from Gene Expression Omnibus (GEO) and used to find genes correlated to γ-catenin expression. There were 42 cell lines shared between the lung cancer cell line datasets (∼60% of the UT study) and both use the same platform (Affymetrix) to assess global gene expression. An absolute r-value of greater than 0.5 (Pearson) was used to define significant correlation to γ-catenin independently in each dataset. There were 324 and 252 transcripts correlated to γ-catenin (r >0.5, Pearson) in the CCLE and UT datasets, respectively, of which 152 were common to both the datasets. We have uploaded an excel spreadsheet as a supplementary table containing the Affy probeset ID and additional annotation for each commonly correlated transcript along with their associated r- and p values for each dataset.

##### Bioinformatic Analysis

The 152 transcripts correlated to γ-catenin were later analyzed for over-representation of biological functions, pathways and connections using the bioinformatics software Ingenuity Pathways Analysis (IPA, www.ingenuity.com). IPA takes a select list of genes and creates limited, interconnected networks (35 genes maximum) based on the evidence of direct or indirect biological relationships contained in the Ingenuity Knowledge Base. The IPA network algorithm seeks to maximize the interconnectivity within a group of selected genes and scores networks based on a right tailed Fisher's exact test that calculates the probability that the given relationships can be explained by a random model. The networks do not include all possible relationships for each member, because of size constraints placed on the network, and specific genes may appear in multiple networks.

##### Data Analysis

Data were compiled from at least three independent, replicate experiments, each performed on separate cultures and on separate occasions. The responses are displayed as “fold-changes.” Comparisons of data among experimental groups were performed using Student's *t* test to assess variance. Increase in statistical significance (*p* value of <0.05) is denoted with an “asterisk” symbol, while a decrease in statistical significance (*p* value of <0.05) is denoted with a “hash” symbol.

## Results

### 

#### 

##### Restoration of γ-Catenin Expression Attenuates NSCLC Cell Migration

**FIGURE 1.**
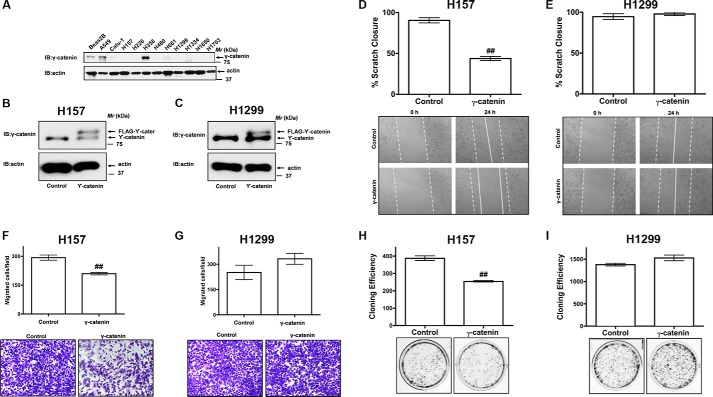
**γ-catenin is a novel regulator of NSCLC cell migration.** *A*, equal amounts of total cell lysates of a non-transformed cell line (Beas2B (B2B)) or NSCLC cell lines (A549, Calu1, H157, H226, H358, H460, H661, H1299, H1334, H1650, and H1703) were separated on SDS-PAGE gels, transferred onto nitrocellulose membranes and the “blots” were later probed with γ-catenin and anti-actin antibodies. *B* and *C*, γ-catenin was transiently expressed in NSCLC cells (*i.e.* H157 and H1299 cells). The cell lysates were later probed to detect the expression of γ-catenin by immunobloting with anti-γ-catenin antibodies. *D* and *E,* a 3-mm scrape wound was created in confluent H157 and H1299 cells transfected with either control or γ-catenin plasmids, and cell migration was recorded at 0 and 24 h as described under “Experimental Procedures.” The *upper panel* represents the quantification of migration as described under “Experimental Procedures,” while representative images were presented in the *lower panel. F* and *G*, migration of H157 and H1299 cells transfected with either control or γ-catenin plasmids were assayed in trans-well inserts as described under “Experimental Procedures.” *Top panel* represents the number of cells migrated, while representative images were displayed in the *bottom panel*. ^##^, *p* < 0.01; *versus* control. *H* and *I*, cell proliferation rates of H157 and H1299 cells transfected with either control or γ-catenin plasmids were determined via clonogenic assays as described under “Experimental Procedures.” *Top panel* represents the cloning efficiency, while representative images were displayed in the *bottom panel*. ^##^, *p* < 0.01; *versus* control.

Plakoglobin (γ-catenin) expression was shown to be either lost or down-regulated in a subset of human lung cancers (22). We further evaluated the expression of γ-catenin in an expanded panel of NSCLC cell lines via immunoblotting with γ-catenin-specific antibodies (Fig. 1*A*) and observed a striking decrease in γ-catenin expression in 82% of NSCLC cell lines tested when compared with a non-transformed bronchial epithelial cell line *i.e.* Beas2B (Fig. 1*A*). However, the role of γ-catenin in NSCLC cell migration has not been completely understood. We chose H157 and H1299 cell lines for our studies since: 1) they express relatively low levels of γ-catenin, and 2) they were amenable to transfections for overexpression and gene knockdown studies. To evaluate the role of γ-catenin in NSCLC cell migration, γ-catenin was expressed in H157 and H1299 cell lines (Fig. 1, *B* and *C*). Following transient transfections, cell migration was assessed via a wound-healing assay (scratch assay) by creating 3 mm wounds on confluent cultures (Fig. 1, *D* and *E*). The abilities of the cells to fill the wound under different treatment conditions were later determined in the presence of a mitosis/cell division inhibitor (mitomycin C). In comparison to control vector transfected cells, H157 cells transfected with γ-catenin displayed reduced cell migration (Fig. 1, *B* and *D*). On the contrary, expression of γ-catenin in H1299 cells failed to reduce cell migration, (Fig. 1, *C* and *E*). To clarify the role of γ-catenin in cell migration, we also performed trans-well migration assays using 8-μm pore size cell culture inserts. Consistent with the scratch assays, expression of γ-catenin in H157 cells, but not in H1299 cells, reduced cell migration (Fig. 1, *F* and *G*). It was interesting to note that H157 and H1299 differ in their p53 mutation status. While, H157 expressed p53 tumor suppressor (harbors a heterozygous non-sense mutation at codon 298), H1299, on the contrary, was p53 null (homozygous partial deletion). Furthermore, the p53-dependent effects of γ-catenin were not restricted to cell migration. γ-catenin also induced strong anti-proliferative effects in H157 cells, but not in H1299 cells, as determined by clonogenic cell proliferation and survival assays (Fig. 1, *H* and *I*). Taken together, these findings suggest that the anti-proliferative and anti-migratory effects of γ-catenin in NSCLC are mediated by p53.

##### Anti-proliferative and Anti-migratory Effects of γ-Catenin in NSCLC Are p53-dependent

**FIGURE 2.**
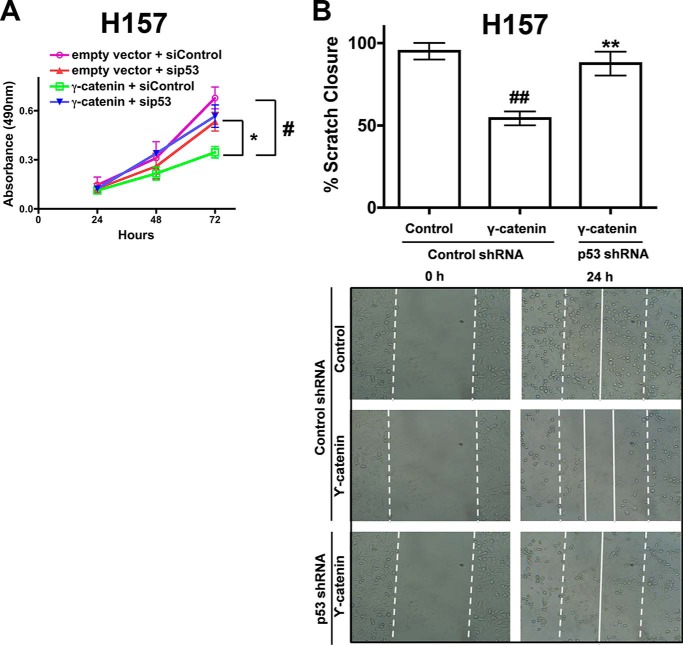
**γ-catenin affects on cell proliferation and migration were p53-dependent.** *A*, H157 cells were co-transfected with γ-catenin plasmids and p53 siRNAs and the proliferation rates of the cells were determined by MTS cell proliferation assays as described under “Experimental Procedures.” Data represent mean ± S.E. from three independent highly reproducible experiments. ^#^, *p* < 0.05; *versus* control. *, *p* < 0.05; *versus* γ-catenin control. *B*, H157 cells were co-transfected with γ-catenin and p53 shRNA expression construct and allowed to grow to confluence. A 3-mm scrape wound was created in confluent cultures, and cell migration was recorded at 0 and 24 h as described under “Experimental Procedures.” The *upper panel* represents the quantification of migration as described under “Experimental Procedures,” while representative images were presented in the *lower panel*.

To further explore the p53-dependent effects of γ-catenin on cell proliferation and cell migration, we utilized short hairpin RNA (shRNA) or short interference RNAs (siRNAs) against p53. Interestingly, expression of γ-catenin failed to induce anti-proliferative (Fig. 2*A*), and anti-migratory (Fig. 2*B*) effects in p53 siRNA or p53 shRNA treated H157 cells, suggesting that the effects of γ-catenin were indeed p53-dependent.

##### γ-Catenin Is a Novel Regulator of p53 Tumor Suppressor Protein

**FIGURE 3.**
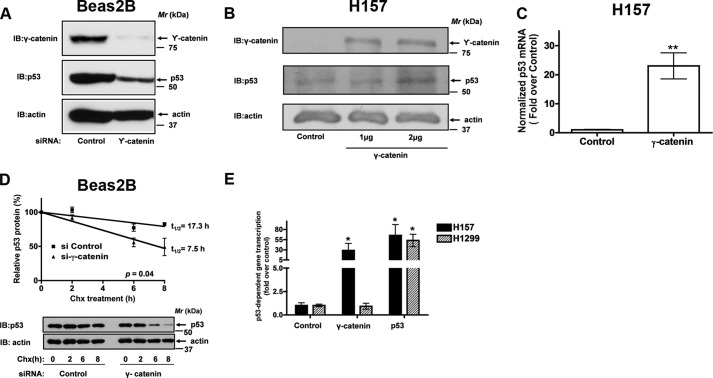
**γ-Catenin is a novel regulator of p53 tumor suppressor protein.** *A*, human non-transformed bronchial epithelial cells (Beas2B) were transfected with either control or γ-catenin siRNAs as described under “Experimental Procedures.” The cell lysates were later probed for the expression of γ-catenin and p53 via immunoblotting with anti-γ-catenin and anti-p53 antibodies. *B*, H157 cells were transfected with either control or γ-catenin plasmids as described under “Experimental Procedures.” The cell lysates were later probed for the expression of γ-catenin and p53 via immunoblotting with anti-γ-catenin and anti-p53 antibodies. *C*, H157 cells were transfected with either control or γ-catenin plasmids as described under “Experimental Procedures.” Total RNAs isolated from the transfected cells were employed in quantitative PCR (qPCR) analysis. **, *p* < 0.01; *versus* control. *D*, Beas2B cells transiently transfected with either control siRNAs or γ-catenin siRNAs were treated with cycloheximide (100 μg/ml) for indicated periods of time, followed by immunoblotting and densitometric scanning. *Upper panel* represents the normalized p53/actin levels, while representative images were displayed in the *lower panel. E*, H157 and H1299 cells were co-transfected either with empty vector or γ-catenin plasmids with p53-luciferase (Stratagene) reporter construct, with luciferase under the control of 14 repeats of p53-binding sequence (TGCCTGGACTTGCCTGG). After 24 h, the cell lysates were assayed for p53-dependent luciferase activities as described under “Experimental Procedures.” Data represent mean ± S.E. of normalized luciferase activities obtained from three independent experiments. *, *p* < 0.05; *versus* control.

We explored next if γ-catenin could regulate p53 expression. To test our hypothesis, we utilized two different strategies: 1) The knockdown of γ-catenin in a non-transformed bronchial epithelial cell line (Beas2B) and 2) The expression of γ-catenin in H157 cells, which have reduced γ-catenin expression (Fig. 1*A*). For knockdown of γ-catenin, siRNAs specifically targeting γ-catenin were designed, tested for their specificity and then employed to selectively suppress γ-catenin. The siRNAs specifically suppressed γ-catenin achieving more than 90% reduction in the expression of γ-catenin (Fig. 3*A*). Interestingly, the suppression of γ-catenin provoked a dramatic loss in the expression of p53 (Fig. 3*A*).

If the hypothesis that γ-catenin mediates p53 expression was correct, expression of a γ-catenin in H157 cells would be expected to induce p53 expression. Transient expression of γ-catenin in H157 cells indeed resulted in an increased expression of p53 (Fig. 3*B*). Regulation of gene expression is mediated by several mechanisms, including transcriptional regulation and/or post-translational regulation. Interestingly, expression of γ-catenin in H157 cells also induced robust p53 mRNA expression, suggesting that γ-catenin might be regulating p53 expression at the transcriptional level (Fig. 3*C*). We also determined whether γ-catenin-mediated p53 expression was due to altered protein stability (Fig. 3*D*). For these studies Beas2B cells were first transfected with either control siRNA or γ-catenin siRNA, followed by the treatment with cycloheximide, which blocks *de novo* protein synthesis. The p53 half-lives were later determined via immunoblotting protein lysates with p53 antibodies (Fig. 3*D*). Interestingly, siRNA mediated knockdown of γ-catenin resulted in the rapid decay of p53 protein in comparison to control siRNA treated cells (Fig. 3*D*). These experiments indicated that γ-catenin also plays an important role in p53 protein stabilization.

We next asked if γ-catenin expression could also induce p53 activation (Fig. 3*E*). For these experiments, we made use of a luciferase reporter vector with luciferase under the control of 14 repeats of p53-binding sequence (Stratagene, tgcctggacttgcctgg). Consistent with the stabilization of p53 (Fig. 3, *B-D*), expression of γ-catenin in H157 induced a 30-fold increase in p53 activity in comparison to control vector transfected cells (Fig. 3*E*). While expression of γ-catenin in a p53 null cell line, H1299, had no effect on p53 activity (Fig. 3*E*). Taken together, these data suggest that γ-catenin is a novel regulator of p53 tumor suppressor protein via transcriptional and post-translational mechanisms.

##### HAI-1/SPINT-1 Is a Novel Downstream Target of γ-Catenin

**FIGURE 4.**
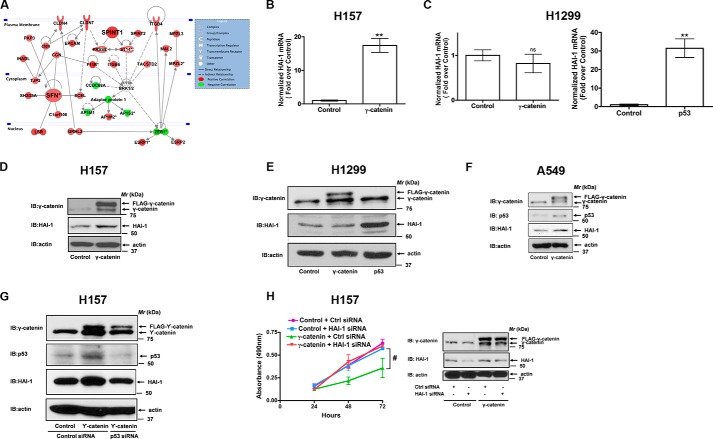
**γ-Catenin is a novel regulator of HAI-1/SPINT-1.** *A*, gene network analysis based on previously known direct (*solid lines*) and indirect (*dashed lines*) biological connections for the identified transcripts with a positive (*red*) and negative (*green*) correlation (r-value >0.5) to γ-catenin expression in two, independent lung cancer cell line datasets. Genes with an *asterisk* were represented more than once in the 152 transcripts correlated to γ-catenin gene expression. *B* and *C*, H157 (*B*) and H1299 (*C*) cells were transfected with either control or γ-catenin plasmids as described under “Experimental Procedures.” Total RNAs isolated from the transfected cells were employed in quantitative PCR (qPCR) analysis. **, *p* < 0.01; *versus* control. *D* and *E*, H157 (*D*) and H1299 (*E*) cells were transfected with either control or γ-catenin plasmids as described under “Experimental Procedures.” The cell lysates were later probed for the expression of γ-catenin and HAI-1 via immunoblotting with anti-γ-catenin and anti-HAI-1 antibodies. *F*, total cell lysates of A549 cells stably expressing γ-catenin were probed for the expression of γ-catenin, p53, and HAI-1 via immunoblotting with anti-γ-catenin, anti-p53, and anti-HAI-1 antibodies. *G*, H157 cells were co-transfected with γ-catenin plasmids and p53 siRNAs as described under “Experimental Procedures.” The cell lysates were later probed for the expression of γ-catenin, p53, and HAI-1 via immunoblotting with specific antibodies. *H*, H157 cells were co-transfected with γ-catenin plasmids and HAI-1 siRNAs as described under “Experimental Procedures,” and their cell proliferation rates were determined with an MTS assay. Data represent mean ± S.E. of three independent experiments. ^#^, *p* < 0.05; *versus* γ-catenin + Ctrl siRNA control.

To investigate the mechanism by which γ-catenin exerts its anti-proliferative and anti-migratory effects, we performed comprehensive pathway analysis and built a gene interaction network based on publicly available gene expression datasets containing an extensive number of lung tumor cell lines. We identified 152 common transcripts, out of 324 and 252 transcripts independently correlated to γ-catenin (r >0.5, Pearson) in two different datasets. There was complete concordance in the direction of correlation between the two datasets, with the tumor suppressor protein, Serine Protease Inhibitor, Kunitz Type 1 (HAI-1 or SPINT1), being the top correlated gene in both datasets (Supplemental Table S1). When we use IPA to connect these genes based on previously known biological associations, a very robust signaling network is produced (*p* = 1 × 10^−70^, Fisher's exact test), with the tumor suppressors HAI-1/SPINT-1 and Stratifin (SFN) central to these connections (Fig. 4*A*).

Having identified this association, we evaluated if γ-catenin could regulate the expression of HAI-1. Interestingly, the expression of γ-catenin in H157 cells induced strong expression of HAI-1 transcripts, as revealed by quantitative PCR (qPCR, Fig. 4*B*). Expression of γ-catenin in H1299 cells, on the contrary, failed to induce HAI-1 mRNA expression (Fig. 4*C*). Although γ-catenin failed to induce HAI-1 mRNA expression in H1299 cells, expression of wild-type p53 cDNAs in H1299 stimulated robust HAI-1 mRNA expression (Fig. 4*C*), suggesting that γ-catenin-induced HAI-1 expression is p53-dependent. Consistent with the effects of γ-catenin expression on HAI-1 mRNA expression, γ-catenin expression also induced HAI-1 protein expression in a p53-dependent fashion (Fig. 4, *D* and *E*). In support of our hypothesis, γ-catenin/p53 regulation of HAI-1 expression was also observed in another NSCLC cell line *i.e.* A549 (Fig. 4*F*). Moreover, γ-catenin-induced HAI-1 expression in H157 cells was also attenuated in the presence of p53 siRNAs (Fig. 4*G*). Of note, *in silico* analysis of HAI-1 promoter for potential transcription factor binding sites also revealed p53 binding sites. Taken together, these data suggest that γ-catenin is a novel regulator of HAI-1 through a p53-dependent mechanism.

To test if the anti-proliferative effects of γ-catenin were mediated via the induction of HAI-1, we have co-transfected H157 cells with γ-catenin and HAI-1 specific siRNAs. Interestingly, γ-catenin-induced anti-proliferative effects were abolished in the presence of HAI-1 siRNAs (Fig. 4*H*), suggesting that the effects of γ-catenin were indeed mediated via the induction of HAI-1.

##### HAI-1 Is a Novel Regulator of Cell Proliferation and Migration in NSCLC

**FIGURE 5.**
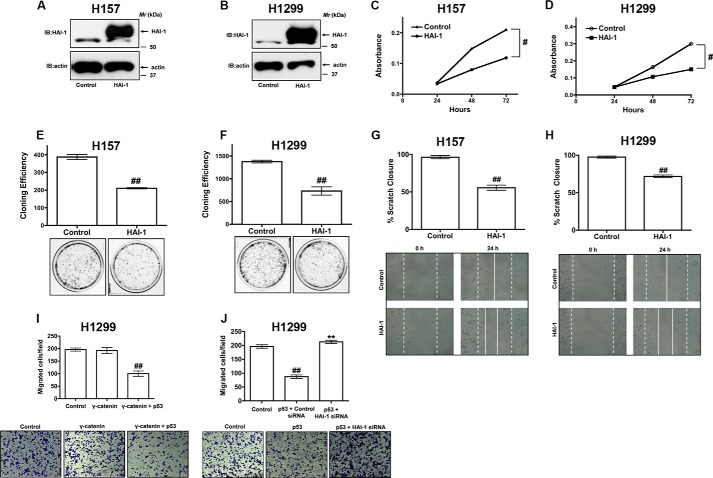
**HAI-1 is a novel regulator of NSCLC cell proliferation and migration.** *A* and *B*, HAI-1 was transiently expressed in NSCLC cells (*i.e.* H157 and H1299 cells). The cell lysates were later probed to detect the expression of HAI-1 by immunobloting with anti-HAI-1 antibodies. *C–F*, cell proliferation rates of H157 (*C* and *E*) and H1299 cells (*D* and *F*) transfected with either control or HAI-1 plasmids were determined with either MTS assays (*C* and *D*) or clonogenic assays (*E* and *F*) as described under “Experimental Procedures.” Data represent mean ± S.E. of three independent experiments. ^##^, *p* < 0.01; ^#^, *p* < 0.05; *versus* control. *G* and *H*, H157 (*G*) and H1299 (*H*) cells were transfected with HAI-1 plasmids and allowed to grow to confluence. A 3-mm scrape wound was created in confluent cultures, and cell migration was recorded at 0 and 24 h as described under “Experimental Procedures.” *I*, H1299 cells were co-transfected with γ-catenin and WT p53 plasmids. Cell migration was later assayed in trans-well inserts as described under “Experimental Procedures.” *Top panel* represents the number of cells migrated, while representative images were displayed in the *bottom panel*. ^##^, *p* < 0.01; *versus* control. *J*, H1299 cells were co-transfected with WT p53 plasmids and HAI-1 siRNAs. Cell migration was later assayed in trans-well inserts as described under “Experimental Procedures.” *Top panel* represents the number of cells migrated, while representative images were displayed in the *bottom panel*. ^##^, *p* < 0.01; *versus* control, **, *p* < 0.01; *versus* p53 + control siRNA control.

HAI-1 was shown to inhibit cell proliferation, migration, and cellular invasion in uterine leiomyosarcoma cells and intestinal tumorigenesis (25, 26). However the role of HAI-1 in NSCLC cell proliferation and migration has not been fully explored. To investigate if HAI-1 could produce similar anti-proliferative effects like that of γ-catenin in NSCLC, we transiently expressed HAI-1 in H157 and H1299 cells (Fig. 5, *A* and *B*). The expression of HAI-1 resulted in reduced cell proliferation rates in H157 cells as determined by an MTS (Fig. 5*C*) and clonogenic cell proliferation assays (Fig. 5*E*). Interestingly, expression of HAI-1 in H1299 cells also affected cell proliferation as determined by an MTS (Fig. 5*D*) and clonogenic assays (Fig. 5*F*), further supporting our earlier assertion that HAI-1 is operating downstream of p53. Furthermore, HAI-1 expression also resulted in reduced cell migration as determined by a scratch assay in both H157 and H1299 cells (Fig. 5, *G* and *H*). Interestingly, expression of WT p53 in H1299 was also able to restore the ability of γ-catenin to inhibit cell migration (Fig. 5*I*). Furthermore, WT p53-induced effects on H1299 cell migration was also HAI-1-dependent (Fig. 5*J*). Taken together, these data suggest novel anti-proliferative and anti-migratory roles for HAI-1 in NSCLC.

##### Expression of γ-Catenin Sensitizes H157 Cells to c-MET Inhibitor

**FIGURE 6.**
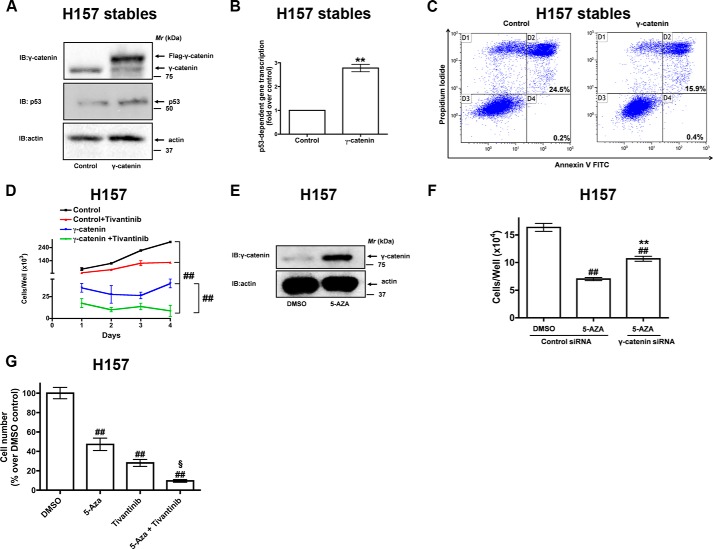
**Expression of γ-catenin sensitizes H157 cells to c-MET inhibitor-mediated growth inhibition.** *A*, lysates of H157 cells stably expressing γ-catenin plasmids were probed for the expression of γ-catenin and p53 via immunoblotting with specific antibodies. *B*, H157 cells stably expressing either control or γ-catenin were transfected with p53-luciferase (Stratagene) reporter construct, with luciferase under the control of 14 repeats of p53-binding sequence (TGCCTGGACTTGCCTGG). After 24 h, the cell lysates were assayed for p53-dependent luciferase activities as described under “Experimental Procedures.” Data represent mean ± S.E. of normalized luciferase activities obtained from three independent experiments. **, *p* < 0.01; *versus* empty vector control. *C*, H157 cells stably expressing either control or γ-catenin were stained for Annexin V/propidium iodide followed by flow cytometry. *D*, H157 cells stably expressing either control or γ-catenin were treated with either DMSO or Tivantinib (0.5 μm) as described under “Experimental Procedures.” The cell proliferation rates were later determined using a hemocytometer. Data represent mean ± S.E. of three independent experiments. ^##^, *p* < 0.01; *versus* control. *E*, H157 cells were treated with either DMSO or 5-Aza (3 μm) for 24 h. The lysates were later probed for the expression of γ-catenin. *F*, H157 cells transiently transfected with either control siRNAs or γ-catenin siRNAs were treated with either DMSO or 5-Aza (3 μm). The cell proliferation rates were later determined using a hemocytometer. Data represent mean ± S.E. of three independent experiments. ^##^, *p* < 0.01; *versus* control siRNA/DMSO control, **, *p* < 0.01; *versus* control siRNA/5-Aza control. *G*, H157 cells were treated with DMSO, 5-Aza (3 μm), Tivantinib (0.5 μm), or 5-Aza and Tivantinib as described under “Experimental Procedures.” The cell proliferation rates were later determined using a hemocytometer. ^##^, *p* < 0.01; *versus* control. ^§^, *p* < 0.01; *versus* 5-Aza or Tivantinib alone.

HAI-1 is known to bind HGFA and repress HGF, the ligand for c-MET receptor signaling (8–14). In this study we have shown that γ-catenin regulates HAI-1 levels (Fig. 4). These findings raise an exciting possibility that γ-catenin expression could be used to sensitize NSCLC cells to c-MET inhibitor. To test this possibility, we made use of H157 cells stably expressing γ-catenin (22). Immunoblotting of total lysates of H157 cells stably expressing γ-catenin revealed a strong expression of γ-catenin (Fig. 6*A*). Stable expression of γ-catenin in H157 also induced p53 expression (Fig. 6*A*) and p53 activation (Fig. 6*B*). Furthermore, stable expression of γ-catenin did not effect cell viability since there was no difference in Annexin V/propidium iodide staining between control cells and H157 cells stably expressing γ-catenin (Fig. 6*C*). As shown earlier (Fig. 1*H*), stable expression of γ-catenin resulted in reduced cell proliferation in H157 cells (Fig. 6*D*). Interestingly, treatment of H157 cells stably expressing γ-catenin with the c-MET inhibitor, Tivantinib, further reduced γ-catenin-mediated growth inhibition (Fig. 6*D*).

Interestingly, treatment of H157 cells with 5-Aza-2′-deoxycytidine (5-Aza, Decitabine), a DNA methyl transferase inhibitor, induced γ-catenin expression (Fig. 6*E*) and reduced cell proliferation (Fig. 6*F*), which concur with our earlier findings (22). Furthermore, 5-Aza-induced effects on cell proliferation were γ-catenin-dependent, since the 5-Aza treatment had reduced effects on H157 cells treated with γ-catenin siRNAs (Fig. 6*F*). We next explored if the treatment of H157 cells with 5-Aza would further sensitize H157 cells to Tivantinib-mediated growth inhibition (Fig. 6*G*). For these experiments, we treated H157 cells with the vehicle (DMSO), 5-Aza, Tivantinib, or with a combination of 5-Aza and Tivantinib (Fig. 6*G*). Notably, treatment of H157 cells with 5-Aza and Tivantinib showed a significant reduction in growth when compared with H157 cells treated with either 5-Aza or Tivantinib alone. In total, these data suggest that the induction of γ-catenin expression, via 5-Aza treatment, might represent a novel strategy to treat NSCLC in combination with c-MET inhibitors.

## Discussion

Plakoglobin (γ-catenin) has a well-defined role as a structural protein (27), where it acts as a scaffold protein in both desmosomes and adherens junctions (20). Interestingly, unlike its closely related homologue β-catenin, γ-catenin was shown to act as a tumor suppressor in NSCLC (22). Re-expression of γ-catenin in NSCLC cell lines resulted in reduced cell proliferation (22). It has also been reported that promoter methylation might be an important cause for the loss of γ-catenin in NSCLC and other carcinomas (22, 28, 29). Moreover treatment of NSCLC cells with a hypomethylating agent 5-aza-2′-deoxycytidine (5-Aza) restored γ-catenin protein levels, suggesting that γ-catenin expression is indeed regulated via promoter methylation (22).

Since the tumor suppressive activities of γ-catenin were apparent (22), we explored next if γ-catenin also played a critical role in cell migration, which is required for tumor progression and metastasis. Consistent with the anti-proliferative role, γ-catenin was also observed to play an important anti-migratory role in NSCLC (Fig. 1). Similar roles for γ-catenin in the regulation of cell migration of human umbilical vascular endothelial (HUVEC) cells (30), MCF7 cells (31), bladder carcinoma (32) and keratinocytes (33) were reported. Moreover, our studies also highlight a novel signal transduction-dependent regulation of cell migration by γ-catenin.

The role of γ-catenin in signal transduction is not fully understood. It has been previously shown that γ-catenin expression in NSCLC cells could reduce the activity of the Lymphoid enhancer factor (LEF)/T-cell factor (TCF)-dependent gene transcription (18, 19, 22). Since β-catenin is a strong inducer of LEF/TCF-dependent gene transcription, it remains unclear, if the γ-catenin affects on LEF/TCF-dependent gene transcription were directly or indirectly associated with the reduction in β-catenin expression. In this study, we have identified a novel role for γ-catenin in the regulation of HAI-1 by a p53-dependent mechanism (Fig. 4). Since p53 mutations in lung cancer are about 50%, restoration of γ-catenin expression as a therapy to target lung cancer tumors that do not express p53 mutants might represent a useful strategy. It was shown earlier that treatment of NSCLC cells with a DNA methyl transferase inhibitor, 5-Aza-2′-deoxycytidine (5-Aza) induced γ-catenin expression (22). Therefore, based on the expression of γ-catenin and p53 mutation statuses it is possible to determine if the solid tumors will be amenable to 5-Aza-based therapy (Fig. 6*G*).

Recent work has also shown that γ-catenin could interact with p53 and induce the gene expression of 14-3-3 (SFN), a tumor suppressor protein in human tongue squamous cell carcinoma SCC9 cells (34). In this study, we show that HAI-1 is a novel down-stream target of γ-catenin in a p53-dependent manner. As identified in SCC9 cells (34), it is possible that the HAI-1 expression could be regulated by the binding of γ-catenin/p53 to the HAI-1 promoter region, or by p53 alone. However, the molecular details of γ-catenin/p53 regulation of HAI-1 remains to be discerned.

The current study also identified HAI-1 as a novel downstream effector of γ-catenin (Fig. 4), most importantly in a p53-dependent manner. It is interesting to note that the phenotype of patients with mutations in γ-catenin (35) were similar to that of mice carrying mutations in HAI-1 (36), characterized by hyperkeratinization of the epidermis. Based on these studies, in tandem with our observations, there is a strong possibility that γ-catenin regulates HAI-1. Additionally, since HAI-1 is a known inhibitor of c-MET signaling, it is also plausible that γ-catenin might indirectly regulate c-MET receptor signaling via the up-regulation of HAI-1 and consequent down-regulation of HGF.

**FIGURE 7.**
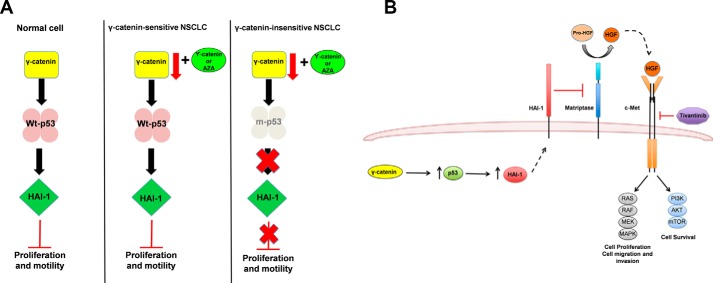
**Models depicting the clinical significance of γ-catenin affects in NSCLC.** *A*, in non-transformed cells, γ-catenin-mediated p53 and HAI-1 expressions are tightly regulated providing controlled cell proliferation and migration. Loss of γ-catenin in NSCLC on the other hand could lead to increased proliferation and migration due to the down-regulation of p53 and HAI-1. While, NSCLCs with wild-type p53 can be sensitized with γ-catenin expression or via 5-Aza treatment, NSCLCs with mutant p53, on the other hand, would be insensitive. *B*, γ-catenin-mediated HAI-1 regulation also offers a promising novel strategy to sensitize NSCLC to c-MET inhibitor-mediated growth inhibition.

The clinical significance of this study is 2-fold: 1) given the regulation of γ-catenin expression via promoter methylation, restoration of γ-catenin expression through the treatment of 5-Aza might be a potential therapeutic option in the treatment of NSCLC. Gauging the expression levels of γ-catenin and p53 mutation status, it is possible to define the sensitivity of NSCLC to 5-Aza treatment (Fig. 7*A*). 2) γ-catenin-mediated HAI-1 regulation also offers a promising novel strategy to inhibit the c-MET signaling pathway (Fig. 7*B*). In addition, the expression levels of γ-catenin and HAI-1 may also serve as novel biomarkers for predicting responses to 5-Aza and/or c-MET inhibitors. Although high doses of 5-Aza showed promise in patients with metastatic lung cancer, low dose 5-Aza was not very effective (15, 37). However, based on our observations a combination of 5-Aza and Tivantinib might be a useful therapy to treat NSCLC.

## Supplementary Material

Supplemental Data

## Footnotes

2 The abbreviations used are:

HGF: hepatocyte growth factor
NSCLC: non-small cell lung cancer
5-Aza: 5-aza-2′-deoxycytidine
LEF: lymphoid enhancer factor
TCF: T-cell factor
shRNA: short hairpin RNA.
