# Supplementary material for: Novel Role for γ-Catenin in the Regulation of Cancer Cell Migration via the Induction of Hepatocyte Growth Factor Activator Inhibitor Type 1 (HAI-1)
Source: J Biol Chem. 2015 Apr 29;290(25):15610–20. doi: 10.1074/jbc.M114.631820 (PMC4505473; doi:10.1074/jbc.M114.631820)
Supplement: Supplemental Data [file supp_290_25_15610__index.html]

Novel Role for γ-Catenin in the Regulation of Cancer Cell Migration via the induction of Hepatocyte Growth Factor Activator Inhibitor Type 1 (HAI-1) — Novel Role for γ-Catenin in the Regulation of Cancer Cell Migration via the Induction of Hepatocyte Growth Factor Activator Inhibitor Type 1 (HAI-1) — γ-Catenin Is a Novel Regulator of HAI-1 — Supplemental Data 

# Novel Role for γ-Catenin in the Regulation of Cancer Cell Migration via the Induction of Hepatocyte Growth Factor Activator Inhibitor Type 1 (HAI-1)

## Supplemental Data

**Files in this Data Supplement:**

- Supplementary table 1 (.xlsx, 70 KB) - Supplementary table 1
